# Immune-related adverse events in checkpoint blockade: Observations from human tissue and therapeutic considerations

**DOI:** 10.3389/fimmu.2023.1122430

**Published:** 2023-01-26

**Authors:** Kristian C. Williams, Abigail Gault, Amy E. Anderson, Christopher J. Stewart, Christopher A. Lamb, R. Ally Speight, Neil Rajan, Ruth Plummer, Arthur G. Pratt

**Affiliations:** ^1^ Translational and Clinical Research Institute, Newcastle University, Newcastle upon Tyne, United Kingdom; ^2^ Northern Centre for Cancer Care, The Newcastle upon Tyne Hospitals NHS Foundation Trust, Newcastle upon Tyne, United Kingdom; ^3^ Department of Gastroenterology, Newcastle upon Tyne Hospitals NHS Foundation Trust, Newcastle upon Tyne, United Kingdom; ^4^ Department of Dermatology, Newcastle upon Tyne Hospitals NHS Foundation Trust, Newcastle upon Tyne, United Kingdom; ^5^ Directorate of Musculoskeletal Services, The Newcastle upon Tyne Hospitals NHS Foundation Trust, Newcastle upon Tyne, United Kingdom

**Keywords:** checkpoint inhibitors (CPI), immune-related adverse events, autoimmunity, dermatological, gastrointestinal, musculoskeletal, pathobiology

## Abstract

Checkpoint inhibitors (CPIs) are monoclonal antibodies which, by disrupting interactions of immune checkpoint molecules with their ligands, block regulatory immune signals otherwise exploited by cancers. Despite revolutionary clinical benefits, CPI use is associated with an array of immune-related adverse events (irAEs) that mirror spontaneous autoreactivity. Severe irAEs necessitate pausing or stopping of CPI therapy and use of corticosteroids and/or other immunomodulatory interventions. Despite increasingly widespread CPI use, irAE pathobiology remains poorly understood; its elucidation may point to targeted mitigation strategies and uncover predictive biomarkers for irAE onset in patients, whilst casting new light on mechanisms of spontaneous immune-mediated disease. This review focuses on common CPI-induced irAEs of the gut, skin and synovial joints, and how these compare to immune-mediated diseases such as ulcerative colitis, vitiligo and inflammatory arthritis. We review current understanding of the immunological changes reported following CPI therapy at the level of peripheral blood and tissue. Many studies highlight dysregulation of cytokines in irAE-affected tissue, particularly IFNγ and TNF. IrAE-affected tissues are also predominantly infiltrated by T-cells, with low B-cell infiltration. Whilst there is variability between studies, patients treated with anti-programmed cell death-1 (PD-1)/PDL-1 therapies seem to exhibit CD8+ T-cell dominance, with CD4+ T-cells dominating in those treated with anti-cytotoxic T-lymphocyte-associated protein 4 (CTLA-4) monotherapy. Interestingly, CD8+CXCR3+ T-cells have been reported to be elevated in gastrointestinal, dermatological and musculoskeletal -irAE affected tissues. These findings may highlight potential opportunities for therapeutic development or re-deployment of existing therapies to prevent and/or improve the outcome of irAEs.

## 1 Introduction

In health, checkpoint molecules, including cytotoxic T-lymphocyte-associated protein 4 (CTLA-4) and programmed cell death-1 (PD-1), become up-regulated following immune activation. Their ligation attenuates immune responses, mitigating unwanted or excessive host tissue damage. Cancers exploit these pathways by upregulating checkpoint molecule expression (1) to promote tumour tolerance. Checkpoint inhibitors (CPI) monoclonal antibodies are a class of drugs that block interactions between checkpoint proteins and their ligands. CPIs have transformed outcomes of many cancers (2) but cause immune-related adverse events (irAEs) of varying severity in up to 95% of patients on combination therapy (3). IrAEs can affect multiple organ systems, including but not limited to the gastrointestinal (GI) tract, skin and musculoskeletal structures; in clinical presentation they often mirror “spontaneous” immune-mediated pathologies of affected tissues. Whilst each irAE type has been linked to improved cancer survival (4–9), the events themselves are often debilitating and rarely life-threatening. IrAE severity is graded using the Common Terminology Criteria for Adverse Events (CTCAE) (10), and current clinical guidelines recommend pausing or stopping CPI therapy for ≥ grade 3 toxicity (3, 11–13). High dose corticosteroids are commonly administered in this setting, but concern about adverse cancer outcomes as a consequence of non-specific immunosuppression persists (14–16). Indeed, prompt and more targeted immunomodulation with biologic drugs may be used to combat irAEs whilst limiting corticosteroid use, but long-term safety data in controlled studies are awaited.

Although generally considered to be driven by adaptive immune mechanisms that recapitulate *intended* consequences of checkpoint inhibition, the precise pathobiology of irAEs remains far from understood. With increasing CPI use (17), the monitoring and treatment of their impacts represents a growing healthcare burden. Identifying robust biomarkers for predicting irAE occurrence in CPI recipients – including their tissue-specificity or responsiveness to targeted intervention(s) – remains a pressing unmet need. Insights gained may furthermore advance understanding of spontaneous immune-mediated disease mechanisms, with far-reaching benefits.

## 2 Gastrointestinal irAEs

### 2.1 Presentation

The commonest GI-irAE symptom is diarrhoea with a median time to onset of 5-8 weeks (18). Potential risk factors, including prior genetic susceptibility and the role of microbial epitopes, remain the subject of investigation. Endoscopy or cross-sectional imaging may demonstrate colitis and/or enteritis which, in more severe cases, mimics the clinical phenotype of ulcerative colitis (UC) (18). The likelihood of these complications and their severity varies with CPI regimen used (**Table 1**), with CTLA-4 recipients experiencing GI toxicity more frequently, and to a higher grade, compared with anti-PD-1/PD-L1 monotherapy recipients (19–22, 29). Other GI-irAEs include gastritis, oesophagitis or duodenitis, with rarer manifestations including chronic pancreatitis, hepatitis and coeliac enteropathy (18, 30). CPI enterocolitis is typically managed with oral or intravenous corticosteroids, escalated where needed to treatment with biologics including the anti-TNF monoclonal antibody infliximab or the gut-selective anti-alpha4beta7 integrin monoclonal antibody vedolizumab (18, 30, 31). However, in as many as a third of patients with diarrhoea, no endoscopic inflammation is seen (32, 33). This may be due to more proximal GI inflammation or an increasingly recognised “CPI-microscopic colitis”, where only histological inflammation is demonstrated. In contrast to the UC phenotype, this presentation is more akin to lymphocytic colitis (34), for which sufferers may benefit from orally administered corticosteroids including the beclomethasone pro-drug “clipper” (35, 36).

**Table 1:** Reported incidences of irAEs from a range of cancer types. Incidences shown in any grade (any), grade 1-2 (G1-2) and grade 3-4 (G3-3).

IrAE type	Anti-CTLA-4			Anti-PD-1			Anti-CTLA-4 & Anti-PD-1		
	Any	G1-2	G3-4	Any	G1-2	G3-4	Any	G1-2	G3-4
Gastrointestinal (diarrhea) (19–22)	9.6-28.0%	17.0-28.0%	1.7-6.0%	8.0-19.0%	19.0%	0.8-3%	16.3-36.0%	36%	1.6-9.6%
Dermatological (rash) (3, 19, 21, 23–26)	26.0-43.5%	13.0-21%	0.1-1.9%	7.6-34.0%	23.0%	0-0.6%	40.3-41.0%	27.0%	4.8-5.0%
Musculoskeletal (arthalgia) (3, 19, 21, 23, 27, 28)	5.0-6.1%	1.0-7.0%	0%	6.0-7.7%	10.0%	0%	10.5-11.0%	13.0%	0.30%

### 2.2 Immune dysregulation

Convincing, if complex, association between the gut microbiome and cancer outcomes in CPI recipients has emerged (37, 38). Moreover, case reports have demonstrated faecal microbiota transplant (FMT) can mitigate both anti-PD-1 tumour-resistance and treatment-refractory GI-irAEs (39, 40), and antibiotic therapy is correlated with high-grade GI-irAE incidence (41) – all consistent with a critical immune-mediating role for the microbiome in this setting that has yet to be properly understood (42).

#### 2.2.1 T-cell dysregulation

In addition to differences in GI-irAE frequency observed between CPI treatments (**Table 1**), distinct immune cell infiltration patterns may further discriminate these pathologies from “spontaneous” inflammatory bowel disease (IBD). Hence, whilst prominent T-cell (but minimal B-cell) infiltration is a general observation in CPI-colitis (43), anti-CLTA-4 therapy has been particularly linked to CD4+ T-cell accumulation and anti-PD-1 with CD8+ T-cell infiltrates (43, 44). The majority of the CD8+CD103+ T-cells in the inflamed gut of CPI-colitis patients were proliferating, whereas active proliferation of CD4+ T-helper 1 (Th1) cells occurred in the peripheral blood (PB), suggesting local and systemic expansion of different T-cell subsets (45). An activated effector population of ICOS+ cells was relatively enriched amongst infiltrating CD4+ T-cells in biopsies from both anti-CTLA-4 and anti-PD1-induced colitis compared with those from IBD patients (44). A landmark study recently used single cell sequencing to compare tissue from melanoma patients who did or did not experience GI-irAEs following various CPI treatments alongside healthy tissue (46). A striking and significant enrichment for CD8+ T-cells exhibiting markers of proliferation (Ki67) and cytotoxicity (granzyme B) was seen in the lamina propria of GI-irAE patients relative to both non-irAE CPI recipients and healthy controls, alongside enhanced IFN response gene expression and a reduction in tissue resident memory (Trm) cells (46). Changes in the CD4+ T-cell compartment in the same tissue included increased frequencies of Th1 cells and increased proportions of both regulatory T-cells (Tregs) and proliferating conventional T-cells, compared with both control groups (46). Anti-CTLA-4 can deplete intra-tumoral Tregs in mice (47) and antibody-dependent cell-mediated cytotoxicity (ADCC)-mediated lysis of Tregs has been proposed as a potential mechanism of irAE development in humans (48). Mirroring other studies, however (44, 49, 50), no diminution, but rather an *increase* of Treg frequency was seen in GI-irAE compared with non-irAE or healthy gut tissue in the study by Luomo et al. (46), although these comparisons lacked a “spontaneous” immune-mediated colitis (IBD) control group. Conversely, Sasson et al. did find Tregs to represent a significantly lower proportion of infiltrating CD4+ T-cells in GI-irAE tissue compared to that of UC during flare (51), and a *functional* reduction of Treg frequency in tissue inflammation that is explicitly CPI-induced could reconcile these apparently conflicting observations.

Mucosal‐associated invariant T (MAIT) cells are innate-like, MHC class I-restricted cells enriched at mucosal sites in health, but which have been shown to accumulate in the inflamed bowel of IBD patients (52). Current studies show MAIT cells are not increased in affected tissues in gut-irAEs but those present express higher levels of granzyme B (46, 51).

#### 2.2.2 Monocytes

Monocytic influx in CPI-colitis tissue has also been reported (45). Interestingly (and in contrast to T-cell infiltrates), monocyte enrichment was found in both inflamed and non-inflamed parts of the gut from these patients compared with healthy control tissue.

#### 2.2.3 Cytokines

Mucosal TNF levels are high in patients with anti-CTLA-4 therapy-induced colitis, with lower levels of TNF associated with response to steroids (44). High levels of TNF signalling specifically in myeloid cells in CPI-colitis patients have also been reported (46). Baseline serum IL-17 also correlates with occurrence of colitis/diarrhoea in CPI recipients receiving anti-CTLA-4 (53) and was elevated in CPI-colitis patients compared to CPI patients who did not have an irAE (54). IL-17 levels in CPI-colitis patients reduced to similar levels to non-irAE patients upon resolution of clinical symptoms. Differences in gene expression of chemokines and their receptors have also been reported in CPI-colitis patients, with higher numbers of T-cells having increased expression of CXCR3 and CXCR6 (46).

## 3 Dermatological irAEs

### 3.1 Presentation

Dermatological toxicities from CPIs have a wide range of clinical presentations including inflammatory eruptions (drug associated maculopapular exanthem, lichenoid reactions, eczematous reactions, psoriasiform reactions and cutaneous sarcoid), vitiligo-like depigmentation rash (VLDR) and life threatening toxic epidermal necrolysis (55–58). Similar to GI-irAEs, dermatological toxicities are commoner in patients on combination CPI therapy, compared to anti-CTLA-4 or anti-PD-1 monotherapy, although grade 3-4 toxicities are rare (**Table 1**) (3, 23–26). Despite dermatological-irAEs being more common with CPI regimens that include anti-CTLA-4, some such as VLDR are particularly associated with anti-PD-1/PD-L1 inhibitors (59). Dermatological-irAEs can differ between cancer types, with VLDR seen more frequently in melanoma patients compared with other malignancies, where it has been associated with favourable prognostic outcomes (60–63).

### 3.2 Immune dysregulation

The frequently observed co-occurrence in immune-mediated skin diseases with those of the GI tract generally, coupled with recently reported links between gut dysbiosis and the development of irAEs (64) (including those of the skin), fuel interest in the concept that a “gut-skin axis” (GSA) (65) may amplify skin pathology in CPI recipients. Immune cell infiltration has been described in case reports of patients with a wide range of dermatological-irAEs including maculopapular eruptions (66), lichenoid reactions (67–70), VLDR (71) and autoimmune skin blistering conditions (bullous pemphigoid) (72, 73)

#### 3.2.1 T-cell dysregulation

In melanoma patients with CPI-induced maculopapular eruptions a preponderance of CD4+ over CD8+ T-cells in cell infiltrates is described, with few B-cells present (66). In CPI-lichenoid reactions, infiltrating T-cells in the epidermis were predominantly CD4+, whilst CD8+ T-cells were located intradermally (67–69). One study of 5 cases of cutaneous lichenoid reactions found that, whilst the dermatopathology bore strong similarity to non-CPI lichenoid lesions, they were distinguished by increased CD163+ histocytes (67). Comparing RNA expression profiles of skin biopsies from anti-PD-1 recipients affected with mainly lichenoid-type irAEs to those derived from a range of non-CPI drug-induced toxic skin rashes, maximal overlap with Stevens-Johnsons syndrome (SJS) and toxic epidermal necrosis (TEN) was observed (70). This transcriptional profile included increased expression of 18 genes including *GZMB, CXCL9 and CXCL10*, seemingly linking PD-1 blockade with activation of cytotoxic T-cell responses in affected tissues. SJS/TEN reactions from CPI therapy can also occur but are extremely rare and it is thought that CD8+ T cells may play a role in the pathogenesis of SJS/TEN in CPI recipients (74–76).

In VDLR, activated CD8+CXCR3+ T-cells are observed in the vitiligo-like infiltrate of anti-PD-1 recipients. CD8+CXCR3+ T-cells may undergo clonal expansion in the periphery followed by migration into skin in dermatological-irAEs (71). CD8+ T-cells from CPI-induced vitiligo differed from healthy controls and spontaneous vitiligo in their abundant production of IFNγ and TNF, and higher circulating levels of the CXCR3 ligand CXCL10 were detected in the serum of these patients, potentially suggesting distinct mechanisms of cytotoxic cell infiltration and damage.

In summary, whilst activated (and, in particular, cytotoxic) T-cells are a dominant feature of dermatological-irAEs generally, with limited skin biopsy data, the diverse phenotypes observed remain largely unexplained. Future work, including comparative evaluations of tissue from larger, well-characterised cohorts using single cell genomics to gain more granular insights, could yet transform understanding of drug-induced and idiopathic immune-mediated skin disease alike.

## 4 Musculoskeletal irAEs

### 4.1 Presentation

The reported incidence of musculoskeletal-irAEs is about 6%, varying between 3.5 and 13.3% (**Table 1**) (3, 23, 27, 28). In the trial setting, CTCAE grade 1-2 adverse events have not always been reported, however, so these figures may still under-represent the overall morbidity burden. In cohorts/case series of such individuals, inflammatory arthritis manifests in over half (77, 78), and may be more likely to be the first irAE presentation in recipients of anti-PD-1/PD-L1 monotherapy (79). We here focus on musculoskeletal-irAEs that cause synovial inflammation, but polymyalgia rheumatica (PMR) syndromes are also common and there is frequently clinical overlap between the presentations (80). Patterns of joint involvement and autoantibody status at presentation are diverse but somewhat divergent from standard rheumatology practice. For example, large joint oligo- and monoarthritis (often involving the knees) seems at least as typical as small joint involvement, with features of seronegative disease including florid tenosynovitis and remitting seronegative symmetric synovitis with pitting oedema (RS3PE) being well-described.

### 4.2 Immune dysregulation

Unlike rheumatoid arthritis (RA), the majority of CPI-induced inflammatory arthritis (CPI-IA) patients are seronegative for circulating anti-citrullinated and/or rheumatoid factor (RF) autoantibodies (78, 80, 81).

#### 4.2.1 T-cell dysregulation

A number of lines of evidence point to dysregulated T-cell homeostasis as a driver of CPI-IA (82–86). Kim et al. recently used a single cell sequencing approach coupled with multi-parameter flow cytometry to compare PB and synovial fluid (SF) cellular compartments of CPI recipients who developed irAEs (82). A wide range of cell subsets were relatively enriched in SF, including proliferating T-cells, an activated, CXCR3hi effector CD8 T-cell subset, Tregs and a Th1 like subset (in anti-PD1 recipients), all recalling observations in GI-irAE tissue described earlier. Enriched IL-17-expressing subsets of both CD4+ and cytotoxic CD8+ T-cells (Th17 and Tc17 cells, respectively) were also observed in this compartment amongst combination CPI recipients, albeit not reaching statistical significance for Tc17s. The latter is nonetheless of interest given recent descriptions of Tc17 expansion in SF of psoriatic arthritis (PsA) patients – another autoantibody seronegative subset of spontaneous inflammatory arthritis, raising the possibility of shared disease pathways between these conditions (87). Two additional cell subsets, MAIT γδ T-cells and a CD38hi CD127- CD8+ effector subset, were relatively enriched in SF – a finding that has since been replicated in a separate study showing these subsets underwent clonal proliferation in the joint and distinguished CPI-IA SF from that of both RA and PsA control patients (86). Diversification of the T-cell repertoire as a consequence of anti-CTLA-4 treatment was previously noted, with more extensive TCR Vβ CDR3 clonotype expansion linked to an increased likelihood of immune-related adverse events in general (88). Sharing of expanded CD8+ clones with CX3CR1hi effector and CXCR3hiCXCR6lo effector phenotypes in the SF and PB of CPI-IA patients, respectively, suggests drug-induced, systemic expansion of CD8 clusters in PB may precede their migration to the joint at irAE onset. Another study also points towards clonal expansion of specific CD8+ T-cells, with some TCR clones being shared across patients (89). Potentially mirroring observations in both “spontaneous” RA and GI-irAEs, current evidence suggests Tregs are not depleted in joints of patients who develop a joint-irAE (82, 85). They are enriched in SF of joint-irAE patients where they are apparently more suppressive than those from PB of both joint-irAE and non-irAE CPI recipients (82).

#### 4.2.2 B-cell dysregulation

B-cells play an undisputed role in the pathogenesis of seropositive RA but, as in the case of GI-irAEs, minimal B-cell infiltration has been reported in the joints of CPI-IA patients, with one report of no detectable B-cells in SF at all (83). However, enhanced systemic B-cell reactivity during CPI treatment amongst melanoma patients who develop irAEs has been observed, with expansion and clonal diversification of a normally rare, CD21^lo^ B-cell population reminiscent of age-associated B-cells whose potential role in RA pathogenesis is under investigation (90). Further work may yet shed light onto their potential relevance in CPI-IA development.

It is important to note that the majority of data arising from joints of CPI-IA patients described here describes characteristics of SF, and not synovial *tissue*. Complimentary and, arguably, more relevant data from a pathobiological perspective is expected to emerge from the study of synovial tissue biopsy samples in the near future (86).

#### 4.2.3 Cytokines

Several cytokines have been reported to be dysregulated in patients with joint-irAEs. TNF has been reported to be highly expressed in joints of joint-irAE patients (83). Meanwhile IL-17 was shown to be elevated in a recurrent case of pseudogout at each flare (85). Other cytokines such as IL-6, IL-1β and IFNγ have also been seen to be increased in SF of joint-irAE patients (82).

## 5 Conclusion and therapeutic considerations

**Figure 1:**
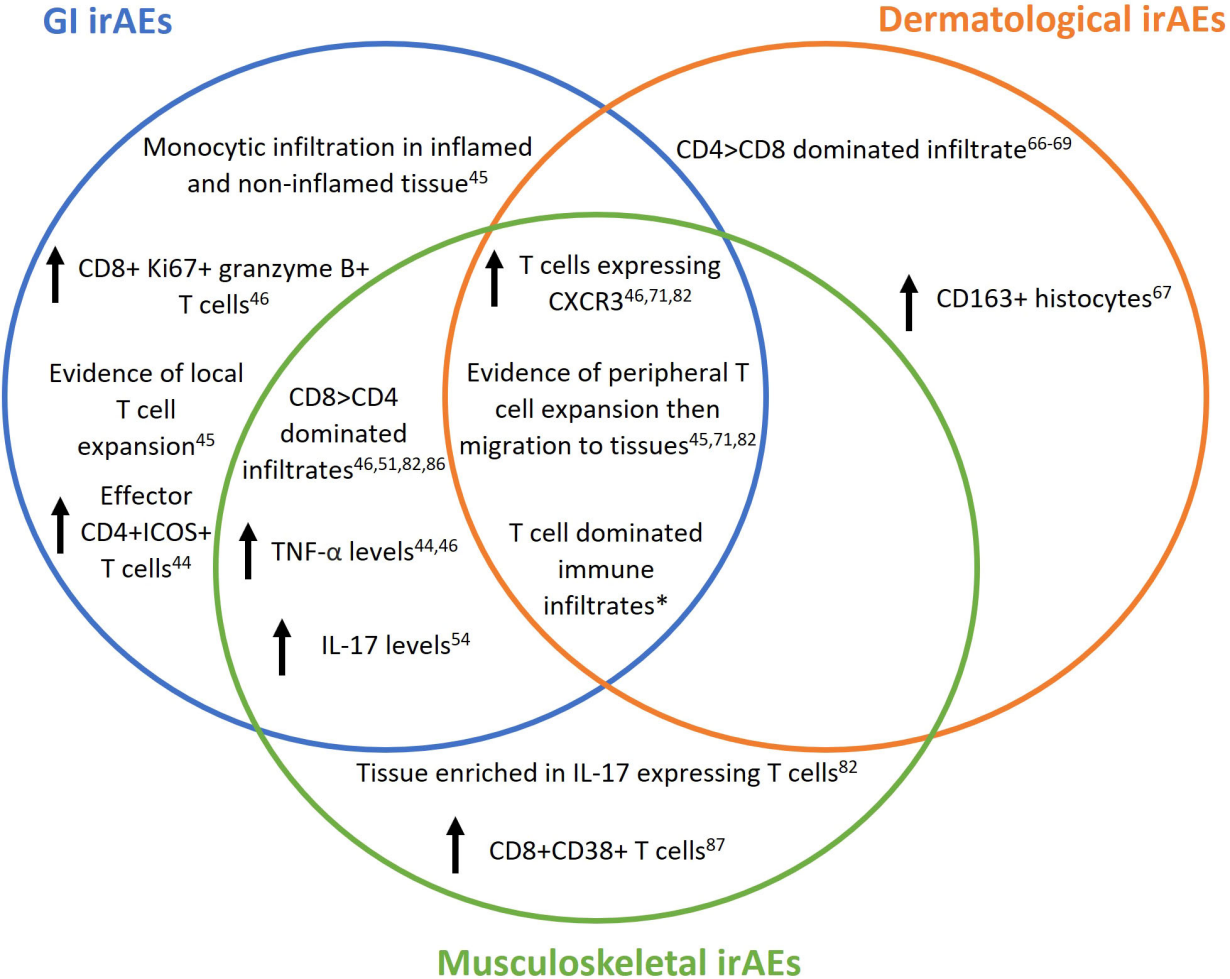
Reported immunological changes in patients on checkpoint inhibitor (CPI) therapy developing immune related adverse events (irAEs). IrAEs are common with CPI use and can affect multiple organs systems including but not limited to gut, skin and joints. Multiple risk factors may contribute to irAE development and/or their localisation to the represented tissues, including genetic variation, microbiome, pre-existing immune-mediated disease and stromal characteristics. Venn diagram depicts immune changes reported in human gut, skin and joint tissue following CPI therapy reported so far in the literature and covered by this review. *All research articles containing peripheral blood or tissue immune characterisation covered in this article.

IrAEs are common, and increasing use of CPIs for an ever-widening range of tumour types and disease stages will inevitably render them more so. Based on disparate studies employing conventional histological techniques, and limited single cell sequencing of disaggregated tissues, certain commonalities and differences between immune dysregulation in dermatological, gastrointestinal and musculoskeletal irAEs are apparent (**Figure 1**). For example, prominent CXCR3+ T-cells (cytotoxic and/or Th1) infiltration appears to be a general feature of affected tissues. Peripheral expansion of T-cells followed by migration into tissues may underpin this facet of irAE development (46, 71, 82) such that enumerating specific PB T-cell subsets could conceivably aid in early irAE detection – a critical consideration for the future. However, as with most putative predictive toxicity biomarkers reported to date, large studies will be needed to validate preliminary observations (91). Mechanisms by which toxicities display varying tissue predilections between individuals remain largely unknown, with genetic variation, the host microbiome, pre-existing immune dysregulation and stromal factors potentially contributing. Beyond irAE development in general, unravelling this complexity could ultimately yield biomarkers that predict tissue specificity and severity. With underpinning data only now beginning to accumulate, we expect this picture to evolve rapidly over the coming years in a fast-moving field that promises important implications for irAE mitigation strategies to support sustained anti-cancer treatment for affected CPI recipients.

Currently, and in keeping with published management guidelines, glucocorticoids remain the first-line therapeutic intervention for moderate to severe irAEs affecting the gut, skin and synovial joint, with doses in excess of 1mg/kg prednisolone advocated in some circumstances (3). The impact of this approach on tumour responses remains unclear: most studies are retrospective such that known association between irAE occurrence and improved tumour outcome may mask any blunting of CPI efficacy as a result of corticosteroid use. On the other hand, whilst the need for irAE treatment with immunosuppression within two months of CPI initiation has been suggested to be deleterious for cancer outcome (92), the confounding impact of premature CPI cessation in this context also remains unclear. The same important caveat applies to data suggesting escalation of immunosuppression, to include targeted interventions including biologic drugs on top of glucocorticoids (93). It should also be remembered that high-dose glucocorticoid use is itself associated with considerable morbidity (94–96). Controlled prospective trials coupled with experimental medicine approaches are needed to properly evaluate these issues, their design informed by emerging pathophysiological insight, should herald more personalised approaches to irAE management (97). For example, clinical trial is currently underway assessing the value of anti-TNFs alongside CPI therapy (NCT03293784).

High levels of proinflammatory cytokines including TNF in irAE-affected gut and synovial tissue suggest strong prima facie rationale for anti-TNF as the most widely deployed targeted therapy for these toxicities (80, 98). The role of this cytokine in tumour biology remains controversial, however (99–101), and some concern about the impact of the approach on cancer outcomes persists. For example, enhanced Treg activity reported with anti-TNF use in RA – of potential concern in the context of a tumour microenvironment – was specifically linked to monoclonal antibody use rather than that of a receptor fusion protein (102), emphasising that the *mechanism of action* of targeted therapy may be *as* important as the molecular targets themselves in this setting. Similar considerations could apply in respect of IL-6 targeting, another increasingly popular approach (103). B-cell depletion with the anti-CD20 monoclonal antibody rituximab has shown some promise for dermatological irAEs, with no apparent adverse impact on survival (104), and observations in inflammatory arthritis reviewed above arguably support interest in the approach. This should, however, be balanced against the general dearth of infiltrating B-cells in irAE tissues described herein, together with very recent data indicating that *regulatory* B-cells (Bregs), which would also be targeted by an anti-CD20 strategy, may play a critical role in the *prevention* of irAEs (105). A deficiency of this cell type in the circulation of CPI recipients was indeed predictive of subsequent toxicity in this study.

Finally, targeting the microbiome of CPI recipients continues to garner interest as a potential route to a more favourable balance between tumour response and irAE development. The use of probiotics as microbial modulators may offer a more streamlined strategy than FMT – which is itself not without risk – but whether the absence of microbial metabolites from such interventions limits efficacy remains to be explored. Multiple early phase trials investigating probiotic supplements alongside CPI therapies are underway, with results set to be available as early as 2024 (106).

In summary, a recent acceleration in studies evaluating irAE target tissue amongst CPI recipients is set to continue, together with data from alternative target tissues not covered in this review, will doubtless inform management strategies of the future. Combined with prospective data from ongoing and planned clinical trials and experimental medicine approaches, these endeavours should yield improved survival outcomes and quality of life for cancer patients.

## Author contributions

All authors contributed to the article and approved the submitted version.
